# Single-cell atlas of human skin implicates APOE pro-inflammatory signaling in diabetic foot ulcers

**DOI:** 10.3389/fimmu.2025.1591944

**Published:** 2025-06-19

**Authors:** Yating Yin, Li Li, Mingen Liu, Bin Wang

**Affiliations:** ^1^ Department of Plastic and Reconstructive Surgery, Shanghai Ninth People’s Hospital, Shanghai Jiao Tong University School of Medicine, Shanghai, China; ^2^ Seventh People's Hospital Affiliated to Shanghai University of Traditional Chinese Medicine, Shanghai, China

**Keywords:** single-cell RNA sequencing, diabetic foot ulcers, apolipoprotein E, human skin fibroblasts, inflammatory disease

## Abstract

**Background:**

Diabetic foot ulcers (DFU) are a major global complication of diabetes mellitus, yet their underlying mechanisms remain incompletely understood. Fibroblasts are key regulators in the finely tuned process of wound healing.

**Methods:**

Single-cell RNA sequencing was performed on human skin tissues to delineate cellular composition and transcriptional profiles.

**Results:**

We identified a distinct fibroblast population overexpressing Apolipoprotein E (APOE) in DFU patients with non-healing wounds. APOE+ fibroblasts were predominantly enriched in DFU patients, and exhibited strong associations with fat cell differentiation and the regulation of epithelial cell proliferation. Metabolic pathway analysis indicated that APOE+ fibroblasts might play a role in the onset and progression of diabetes through the Drug Metabolism-Cytochrome P450 pathway. Pseudotime analysis suggested that APOE+ fibroblasts are in an intermediate differentiation state. CellChat analysis highlighted the significant role of the FGF signaling pathway in DFU. Immunohistochemical staining confirmed upregulated APOE expression in DFU tissues. Ex vivo experiments demonstrated that soluble APOE accelerated fibrosis and inflammation in human fibroblasts, suggesting its detrimental role. Furthermore, high glucose elevated APOE expression and induced a profibrotic and inflammatory phenotype in human fibroblasts.

**Conclusions:**

This study provides critical insights into the differences between healthy and DFU fibroblasts, identifying specific cell populations that may influence DFU healing. These findings may contribute to future therapeutic development for DFU.

## 1 Introduction

Diabetic foot ulcers (DFU) are a severe complication of diabetes mellitus (DM), leading to functional decline, increased infection risk, hospitalization, lower-extremity amputation, and mortality (1). Globally, an estimated 9.1 to 26.1 million individuals with diabetes develop foot ulcers annually (2). Patients with DFU face a five-year mortality risk 2.5 times higher than those without ulcers (3). Notably, over 50% of patients undergoing amputation due to DFU die within five years, a mortality rate exceeding that of many cancers (4). The direct costs of treating DFU surpass those of several common cancers (5). As the prevalence of DM rises, DFU will impose an escalating financial burden on global healthcare systems, potentially becoming one of the most expensive diabetes-related complications (6).

The pathophysiology of DFU involves a complex interplay of multiple factors, primarily including peripheral neuropathy, peripheral arterial disease, and impaired wound healing. In diabetic patients, impaired wound healing often results in chronic foot wounds, especially when neuropathy and/or vascular disease are present (2). Effective wound healing requires a tightly regulated process involving diverse cells and mediators, including platelets, coagulation factors, immune cells, and structural cells (7). Fibroblasts, stromal cells distributed throughout nearly all organs and tissues, exhibit multiple cell clusters and heightened inflammation in the dorsal skin of DM and DFU patients, likely due to diabetes-associated low-grade chronic inflammation (8). Recent studies emphasize their phenotypic plasticity in response to tissue injury and their dynamic role in maintaining tissue homeostasis and integrity (9). However, their specific contributions to impaired healing in DFU remain poorly understood. Comparing cellular differences between DFU patients with healing versus non-healing ulcers, as well as between DM patients and non-DM healthy controls, may provide new insights into DFU pathogenesis.

Single-cell RNA sequencing (scRNA-seq) is a powerful tool for exploring cell function and disease mechanisms by profiling the transcriptomes of individual cells within heterogeneous tissues (10). In this study, we focused on comparing DFU patients with healthy donors, hypothesizing that diabetic patients with impaired wound healing exhibit aberrant gene and protein expression profiles driving fibrosis and inflammation. To investigate this, we conducted scRNA-seq analysis of DFU and healthy skin biopsies to identify molecular changes. We also conducted immunostaining on DFU and healthy skin samples and validated key findings through ex vivo experiments. These results provide a valuable resource for understanding fibroblast roles in DFU and offer insights into developing pro-inflammatory fibroblast-based immunotherapies.

## 2 Results

### 2.1 ScRNA sequencing reveals major cell types during DFU progression

**Figure 1:**
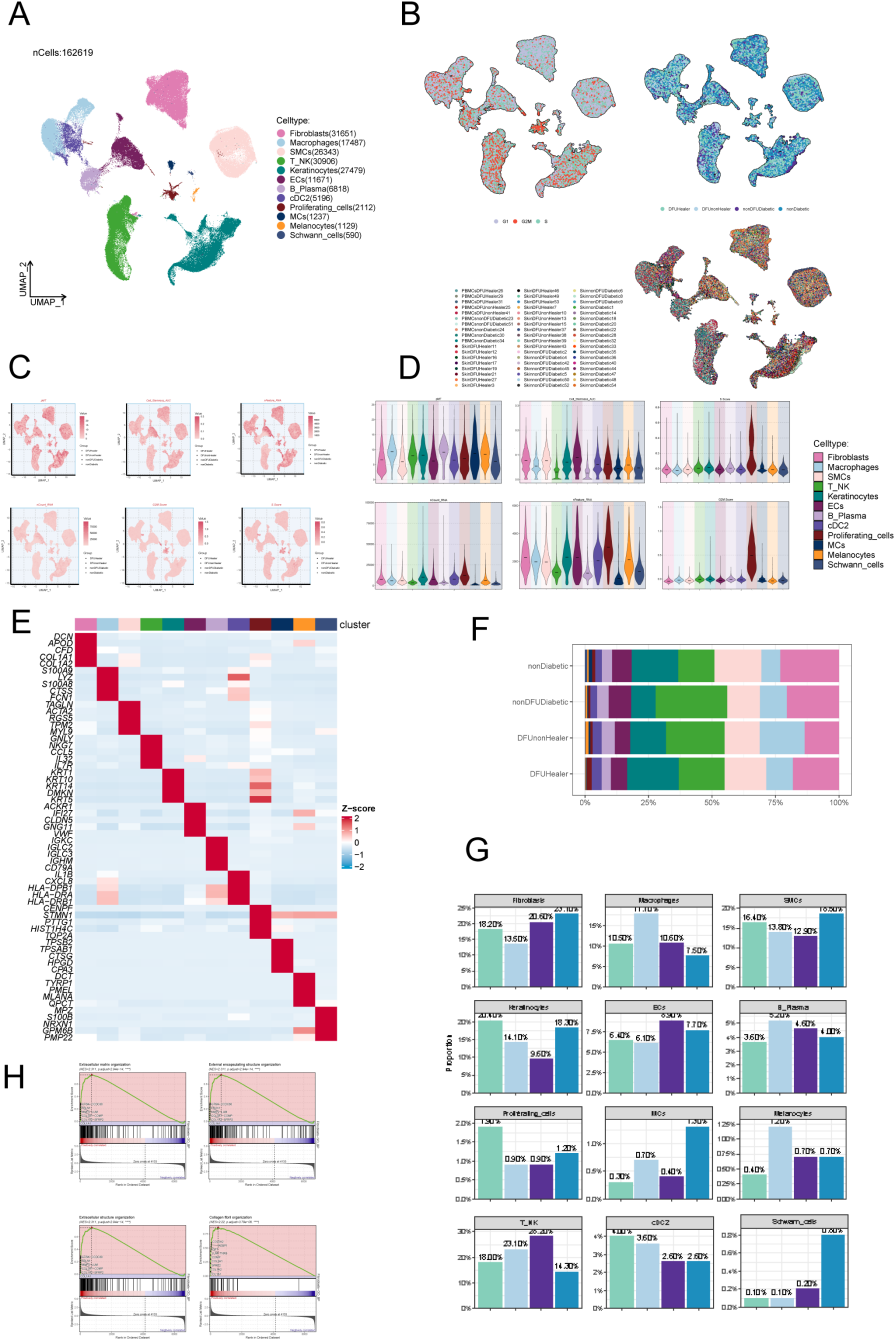
ScRNA sequencing revealed major cell types during DFU progression. **(A)** UMAP plots depicting all cellular subpopulations across four groups: diabetic foot patients, diabetic foot recovery patients, diabetic patients without foot ulcers, and healthy individuals, highlighting differences in cellular composition during disease progression. **(B)** UMAP plots showing the distribution of cell subpopulations according to different cell cycle stages and tissue sources, demonstrating variability in proliferation status across origins. **(C)** UMAP plots illustrating AUCell scores for all subpopulations, representing gene set activity in individual cells. **(D)** Violin plots displaying AUCell scores across all subpopulations, enabling comparison of pathway or gene set activity. **(E)** Heatmap highlighting the top expressed genes in each subpopulation, identifying characteristic gene expression patterns. **(F)** Proportional composition of cell subtypes across four sample groups: nonDiabetic, nonDFUDiabetic, DFUHealer, and DFUnonHealer. Each stacked bar represented the relative abundance of identified cell subpopulations within each group. Statistical comparisons of cell-type proportions between groups were performed using Chi-square test. *p* < 0.05 was considered statistically significant. **(G)** Proportions of cell types in different groups (mean ± SD). Statistical significance between groups was determined by the chi-square test, with all pairwise comparisons showing significant differences (*p*<0.0001). **(H)** GSEA analysis results for fibroblast subpopulations, indicating enriched biological functions and pathways.

To investigate cellular heterogeneity in diabetic foot, we analysed the scRNA-seq data of skin tissue samples of 7 DFU patients, 12 recovered DFU patients, 15 healthy individuals, and 10 diabetic patients without DFU. After applying quality control and filtering, the gene expression of 162,619 individual cells was analyzed. Dimensionality reduction and clustering identified twelve distinct cell types (**Figure 1A**): fibroblasts (n=31,651); macrophages (n=17,487); smooth muscle cells (SMCs, n=26,343); T-NK cells (n=30,906); keratinocytes (n=27,479); endothelial cells (ECs, n=11,671); B-plasma cells (n=6,818); cDC2 (n=5,196); proliferating cells (n=2,112); mast cells (MCs, n=1,237); melanocytes (n=1,129); schwann cells (n=590). Cell origins included 48,835 from recovered DFU patients, 30,709 from DFU patients, 22,631 from diabetic patients without DFU, and 60,444 from healthy controls (**Figure 1B**, right). Cell cycle analysis revealed distributions in S phase (n=57,962), G1 phase (n=72,545), and G2M phase (n=32,112) (**Figure 1B**, left). UMAP and bar plots visualized several key parameters for all cells, including the PMT scores, AUCell scores, nFeature RNA, nCount RNA, G2M scores, and S scores (**Figures 1C-E**). Fibroblast subpopulations were detected in all four groups (**Figure 1F**). Furthermore, distribution analysis showed fibroblast proportions were significantly lower in DFU patients compared to other groups (**Figure 1G**, **Supplementary Figure S1**). Finally, GSEA enrichment analysis highlighted fibroblast involvement in extracellular matrix organization, encapsulating structure organization, collagen fibril organization, and extracellular structure organization (**Figure 1H**).

### 2.2 DFU healing is significantly associated with a specific subset of fibroblasts.

**Figure 2:**
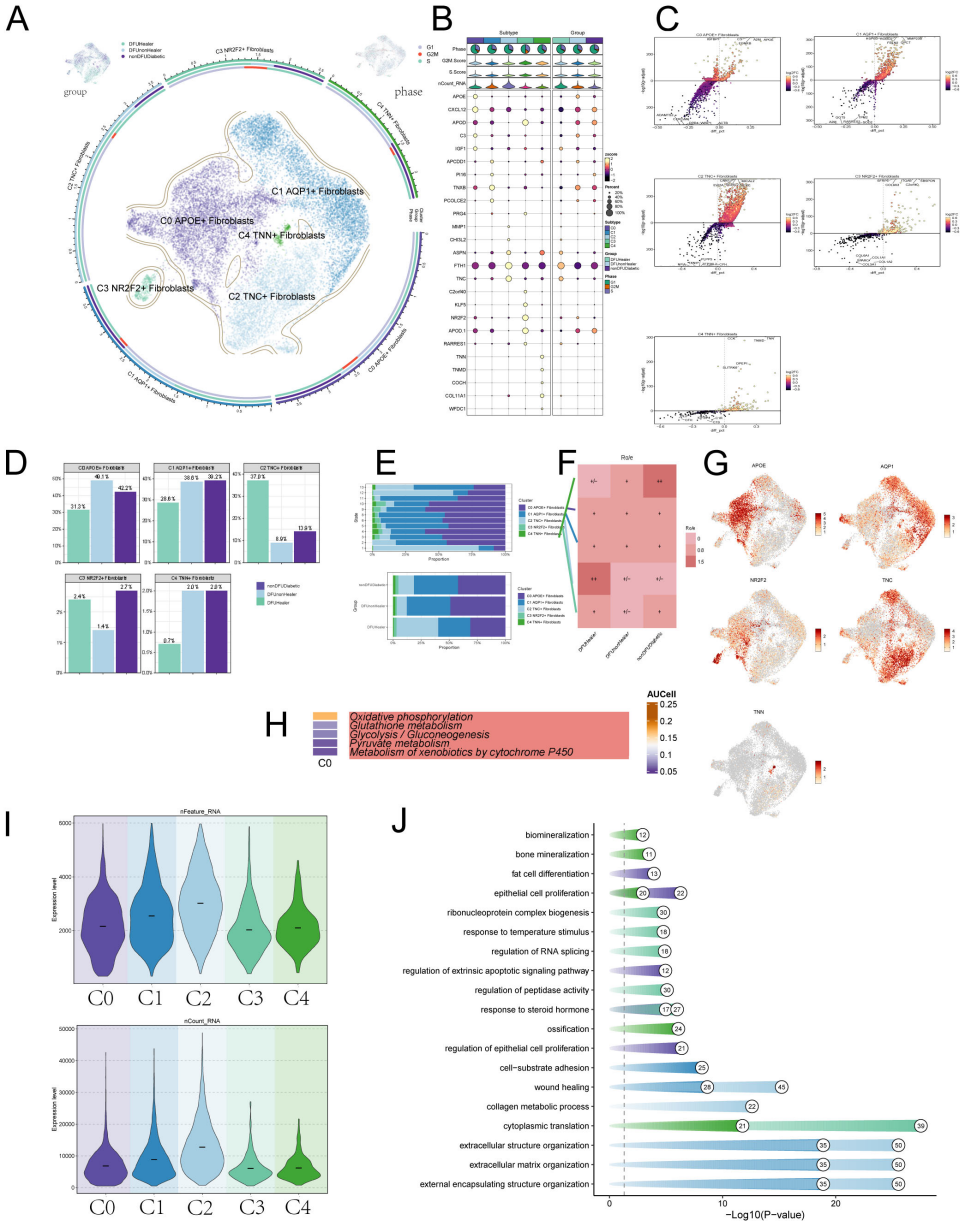
Visualization of DFU patient fibroblasts subpoplulations. **(A)** The UMAP plots illustrate the distribution of cells from different tissue sources—including DFU recovery patients, non-recovered DFU patients, and non-DFU diabetic patients (top left); cell cycle phase distribution (top right); and the distribution of fibroblast subpopulations (center), collectively revealing dynamic changes in subpopulation composition across different conditions. **(B)** Bubble plot showcasing marker gene expression across subpopulations and tissue origins, with bubble size indicating expression percentage and color indicating Zscore. **(C)** Volcano plots displaying differentially expressed genes in five fibroblast subpopulations, identifying subgroup-specific signatures. **(D)** This bar charts compares the distribution of fibroblast subsets under different treatment conditions. Statistical significance between groups was determined by the chi-square test, with all pairwise comparisons showing significant differences (*p*<0.0001). **(E)** Bar charts depicting fibroblast subpopulation expression levels by cell cycle stage and tissue origin. Statistical significance between groups was determined by the chi-square test, with all pairwise comparisons showing significant differences (*p*<0.0001). **(F)** Role analysis plots further detailing fibroblast subpopulation expression across tissue origins. **(G)** UMAP plots showing marker gene expression in fibroblast subpopulations. **(H)** Top five metabolism-related activities in the C0 fibroblast subpopulation. **(I)** Violin plots illustrating RNA expression across fibroblast cell subpopulations. **(J)** Dot-line graphs presenting metabolic activities across different cellular subpopulations.

After excluding data from healthy controls, we applied the inferCNV algorithm to perform sub-clustering analysis of fibroblasts in tissue samples from diabetic patients, recovered DFU patients, and diabetic patients without DFU, identifying five distinct fibroblast subpopulations. Fibroblasts were classified primarily based on their marker genes: C0 APOE+ fibroblasts; C1 AQP1+ fibroblasts; C2 TNC+ fibroblasts; C3 NR2F2+ fibroblasts; C4 TNN+ fibroblasts (**Figure 2A**). A bubble chart visualized marker gene expression for each subcluster (**Figure 2B**). Subsequently, we used volcano plots to depict distinct gene expression patterns across the five subclusters. We found that the differentially expressed genes significantly upregulated in the C0 subcluster include APOE, A2M, C3, IGFBP, and EGNRB (**Figure 2C**). Bar charts illustrated the distribution of fibroblast subclusters across tissue origins and differentiation stages. C0 was predominantly expressed in diabetic foot patients, C1 was highly expressed in both diabetic foot and diabetic patients without diabetic foot, C2 was primarily expressed in recovered diabetic foot patients, and C3 and C4 showed lower expression levels (**Figures 2D, E**). Ro/e analysis (**Figure 2F**) and UMAP plots (**Figure 2G**) further visualized the distribution of these subpopulations, revealing that C2 was most highly expressed in recovered diabetic foot patients (DFUHealer), while C4 was most highly expressed in diabetic patients without diabetic foot (nonDFUDiabetic). We then conducted metabolic pathway analysis for the C0 subpopulation, which showed strong associations with oxidative phosphorylation, Glutathione metabolism and Glycolysis / Gluconeogenesis (**Figure 2H**). We also presented violin plots describing the nFeature-RNA and nCount-RNA values for the five fibroblast subpopulations (**Figure 2I**) and depicted the cellular activities associated with these subpopulations (**Figure 2J**). Notably, the C0 subpopulation exhibited close associations with fat cell differentiation, epithelial cell proliferation, and regulation of epithelial cell proliferation.

### 2.3 APOE+ fibroblasts influence the onset and progression of diabetes through the Drug Metabolism–Cytochrome P450 pathway

**Figure 3:**
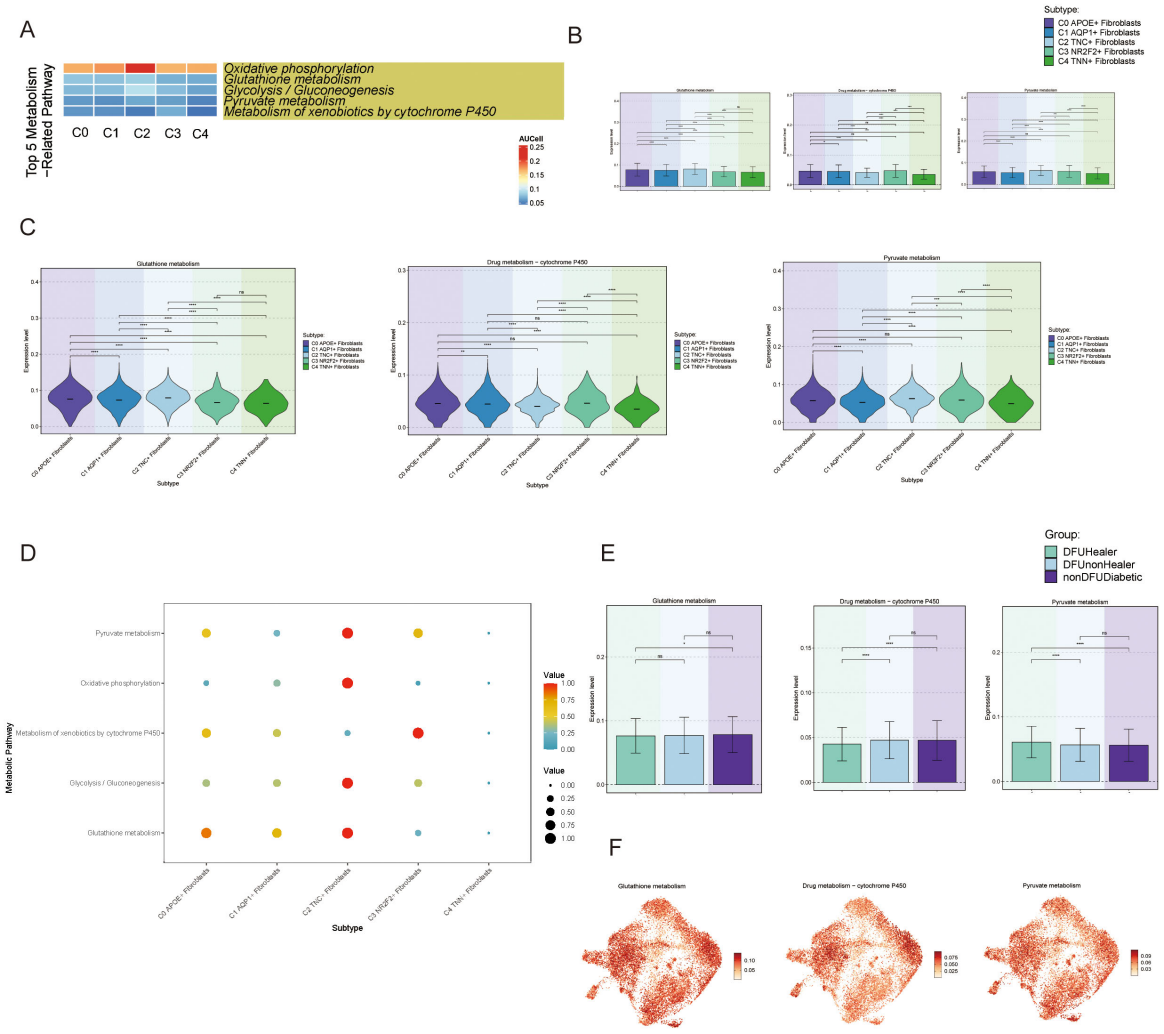
Activity of metabolism-related pathways in DFU cells **(A)** Heatmaps showing the top five enriched metabolism-related pathways in fibroblast subpopulations (C0-C4), highlighting the metabolic heterogeneity across subpopulations. **(B)** Bar charts comparing the relative activity levels of the top five metabolism-related pathways in C0-C4 fibroblasts, highlighting the distinct metabolic programs characteristic of each subpopulation. Statistical significance between groups was assessed by chi-square test (*p*<0.0001, ns = not significant). **(C)** Violin plots further illustrate the expression distribution of genes involved in representative metabolic pathways across fibroblast subpopulations. **(D)** Bubble plots presenting pathway activity scores for each fibroblast subpopulation, **(E)** Bar charts showing differential enrichment of the top metabolic pathways in fibroblast subpopulations across tissue origins, suggesting environmental influence on metabolism. **(F)** UMAP plots demonstrating spatial expression patterns of key metabolic genes from top enriched pathways in fibroblast subpopulations, indicating cellular-level metabolic specialization.

Next, we explored metabolism-related signaling pathways. We first presented the top five metabolic pathways in the fibroblast subpopulations (**Figure 3A**) and focused on three metabolic pathways more closely related to the C0 subpopulation: Glutathione Metabolism and Pyruvate. We displayed the expression levels of these pathways in the five fibroblast subpopulations (**Figures 3B-D**) and compared the metabolic expression levels across different tissue sources (**Figures 3E, F**). We observed that the Drug Metabolism–Cytochrome P450 pathway was lower in tissue samples from recovered diabetic foot patients compared to the disease group, suggesting that the C0 and C1 subpopulation might influence the onset and progression of diabetes through this metabolic pathway. Studies have also shown a connection between APOE and the cytochrome P450 pathway, which is consistent with our findings (11).

### 2.4 APOE+ fibroblasts in DFU influence the differentiation process and exhibited significant importance in the FGF signaling pathway

**Figure 4:**
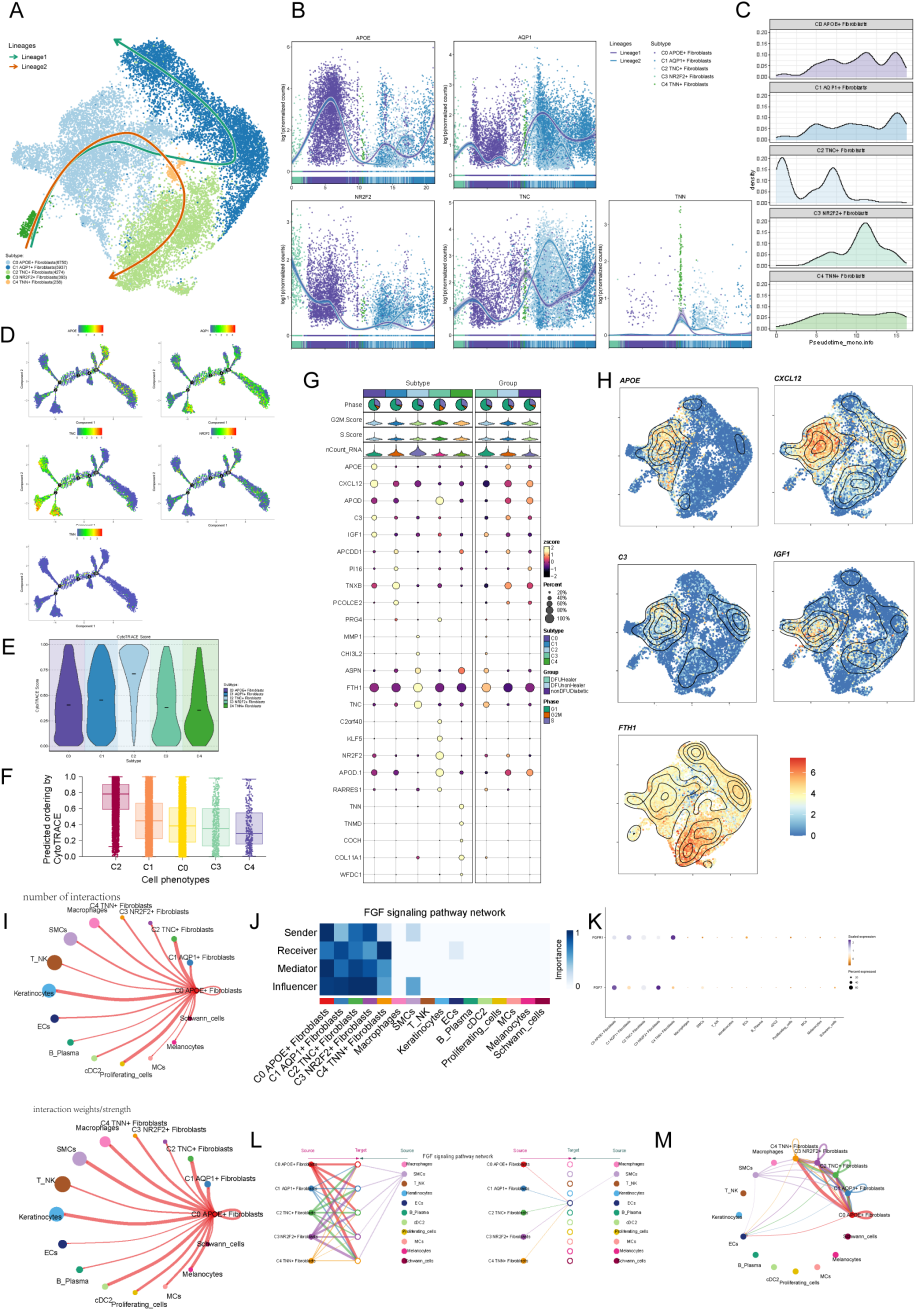
Visualization of pseudotime analysis and cellchat analysis among cells. **(A)** Pseudotime analysis using slingshot demonstrates the inferred differentiation trajectory of fibroblast subpopulations (C0–C4), revealing potential lineage relationships and transition states. **(B)** Pseudotime trajectories of fibroblast subpopulations specifically in diabetic foot patients illustrate their developmental progression within the disease context. **(C)** Ridge plots show the distribution of pseudotime scores among C0–C4 fibroblasts in DFU samples, identifying C0 cells as an early-stage population. **(D)** Detailed Monocle trajectory plot displaying branch points and transcriptional bifurcation events in fibroblast subpopulations, suggesting functional divergence. **(E)** Violin plots of CytoTRACE scores depict differentiation potential of fibroblast subpopulations, with higher scores indicating less differentiation. **(F)** Boxplots comparing CytoTRACE scores among fibroblast subpopulations confirm C0 as a progenitor-like population. **(G)** Bubble plots present the top five marker genes for each fibroblast subpopulation, reflecting their molecular identity. **(H)** UMAP plots visualizing the expression of these marker genes in spatial context. **(I)** Circular plots generated by CellChat reveal the strength and frequency of communication signals sent by each fibroblast subpopulation. **(J)** Heatmap illustrating fibroblast involvement in the FGF signaling pathway, which is known to promote angiogenesis and wound healing. **(K)** Network centrality scores show the dominant signaling hubs within the fibroblast population in the FGF pathway. **(L)** Hierarchical diagram summarizing the receiver strength of each subpopulation within the FGF signaling cascade. **(M)** Circular plots showing fibroblast subpopulations as FGF signal receivers, emphasizing the role of APOE+ fibroblasts (C0) in FGF-mediated communication.

To infer the dynamic changes and potential differentiation trajectories of cells during development, we employed two widely used trajectory inference tools: CytoTRACE and Slingshot. CytoTRACE is based on the principle of transcriptional diversity in single cells, operating under the assumption that cells with higher transcriptional diversity are more likely to reside in an earlier developmental state. This method does not rely on prior temporal information, making it particularly suitable for identifying stem-like cells or potential differentiation origins. Slingshot, on the other hand, constructs potential lineage trajectories by combining unsupervised clustering with low-dimensional embeddings (e.g., UMAP), and infers developmental paths using a minimum spanning tree (MST) algorithm followed by pseudotime scoring for individual cells. These two methods are conceptually complementary. We utilized both to cross-validate the inferred lineage trajectories, thereby increasing the robustness and reliability of our developmental inference. The results from Slingshotreveal two main lineages. The first lineage progressed from C3 → C0 → C4 → C1, and the second lineage from C3 → C0 → C4 → C2 (**Figure 4A**). We further illustrated the expression of marker genes for the five fibroblast subpopulations during pseudotime, finding that the C0 subpopulation marker gene APOE+ was expressed at higher levels in the later stages (**Figures 4B-D**). Cytotrace analysis (**Figures 4E, F**) revealed that the C2 subpopulation exhibited high cellular pluripotency, while the C0 subpopulation was in an intermediate state of differentiation, suggesting that C0 may influence the differentiation process of fibroblasts. Finally, we analyzed the top five genes for each subpopulation (**Figure 4G**), finding that C0 highly expressed APOE, CXCL12, APOD, C3, IGF1, and FTH1, which we visualized using UMAP plots (**Figure 4H**).

To systematically explore complex cellular responses, we investigated intercellular relationships and ligand-receptor communication networks using CellChat analysis. We constructed an initial communication network among cell types, including B lymphocytes, T cells, cDCs, and others, quantifying interaction frequency (represented by line thickness) and intensity (represented by line count) (**Figure 4I**). To identify key incoming and outgoing signals for the five fibroblast subpopulations, we applied CellChat’s pattern recognition method to evaluate ligand-receptor networks. In diabetic foot, cells act as both signal senders (releasing cytokines/ligands) and receivers (responding to ligands), with intercellular communication likely driving disease progression. We visualized the FGF signaling pathway (**Figure 4J**) and analyzed the FGF signaling pathway (**Figure 4K**), highlighting the prominence of the C0 APOE+ fibroblast subpopulation. Centrality metrics identified the C0 APOE+ fibroblast subpopulation as a key mediator and influencer in the FGF signaling pathway. Interaction hierarchy maps revealed fibroblast subpopulations targeting smooth muscle cells (SMCs), and strong fibroblast-keratinocyte interactions (**Figure 4L**). These findings suggest that all fibroblast subtypes may participate in the FGF signaling pathway, with the C0 APOE+ fibroblast subpopulation playing the most significant role (**Figure 4M**).

### 2.5 APOE+ fibroblast subpopulation is more closely associated with the pathogenesis of DFU

**Figure 5:**
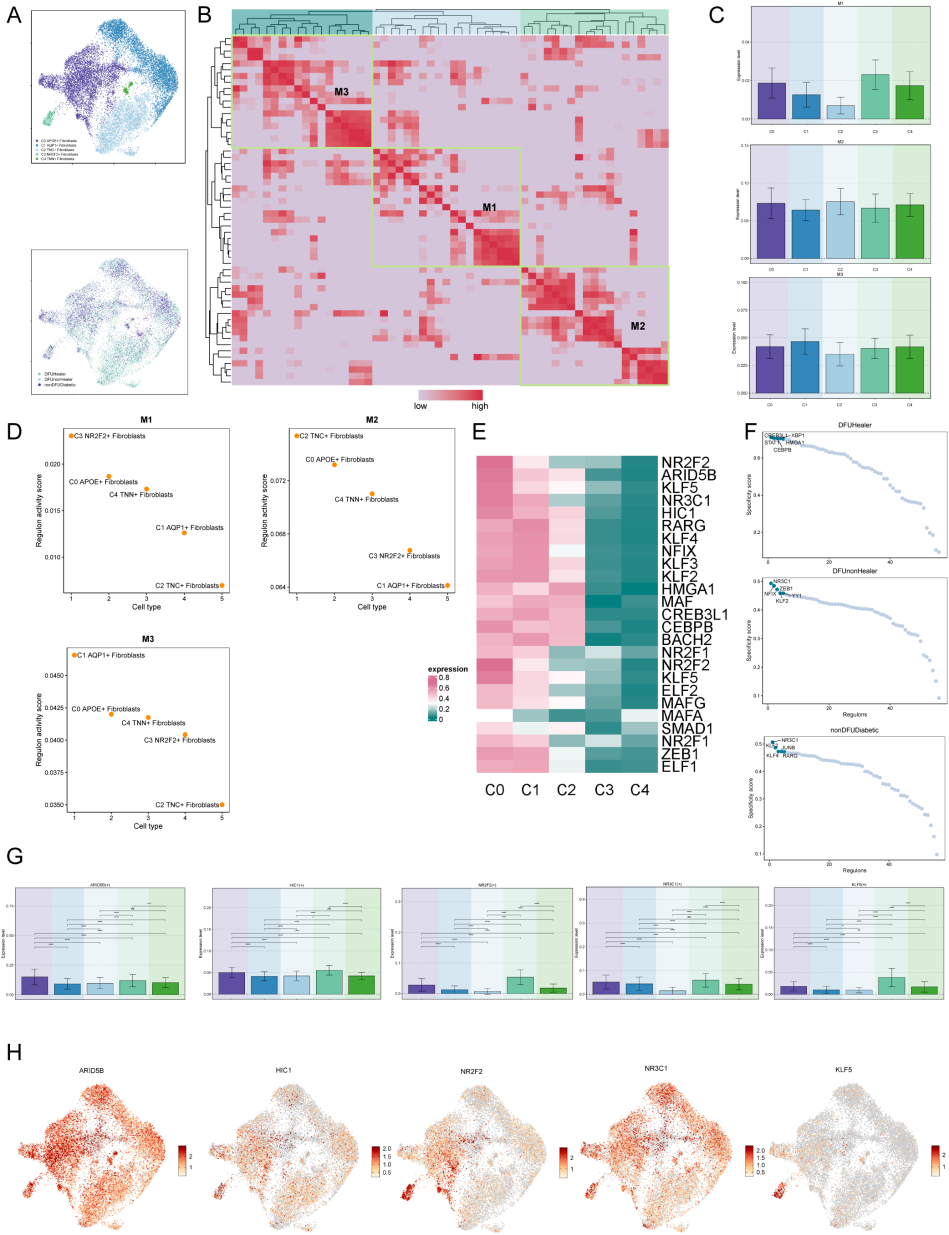
Visualization of expression level of transcription factors of diabetic foot cells. **(A)** UMAP plots showing the distribution of fibroblast subpopulations and the relative expression of transcription factors across tissue origins. **(B)** Non-negative matrix factorization (NMF) clustering identifies three co-expression modules (M1, M2, M3) among fibroblast subpopulations. **(C)** Bar plots illustrate expression levels of M1–M3 modules across fibroblast subpopulations, suggesting functional divergence. **(D)** Regulon activity scores of six fibroblast subpopulations across functional modules M1-M3. **(E)** Heatmaps of these top marker genes demonstrate their co-regulated patterns and distinguish functional identities of each module. **(F)** Scatter plots further show how marker gene expression differs by tissue origin, highlighting environmental effects on transcriptional programs. **(G)** Bar charts comparing the expression of top five transcription factors in C0 fibroblasts with other subpopulations. **(H)** UMAP plots visualize the expression of these transcription factors across fibroblast subpopulations, indicating their spatial regulation.

Finally, we employed UMAP plots to illustrate the expression profiles of different fibroblast subpopulations and their tissue origins (**Figure 5A**). Subsequently, we performed clustering analysis using non-negative matrix factorization (NMF), identifying three distinct clusters (**Figure 5B**). The NMF clustering analysis was conducted to identify distinct gene regulatory modules within the fibroblast populations. The M1, M2, and M3 clusters were defined based on the activity scores of regulons derived from transcription factor analysis, with each cluster representing a set of co-regulated genes showing distinct expression patterns. The selection of these clusters was driven by clear differences in regulon activity profiles observed in the data. Using bar and scatter plots, we visualized the expression patterns of various fibroblast subpopulations within M1, M2, and M3 modules (**Figures 5C, D**). Notably, the C0 subpopulation exhibited relatively high expression of genes associated with the M2 cluster, consistent with increased expression observed in diabetic foot samples. However, we acknowledge that **Figure 5D** shows the C2 subpopulation has the highest M2 regulon activity score. Despite this, APOE expression is particularly enriched in C0 cells and has been functionally validated to play a critical role in diabetic foot pathogenesis, supporting its importance as a key gene within this subpopulation.

Next, we generated a heatmap to depict the top five transcription factors in each fibroblast subpopulation (**Figure 5E**) and utilized scatter plots to present the top five transcription factors of fibroblast subpopulations based on tissue origin (**Figure 5F**). Finally, bar charts and UMAP plots were utilized to describe the expression of the top five transcription factors of the C0 subcluster across different fibroblast subclusters (**Figures 5G, H**).

### 2.6 APOE+ fibroblasts promote fibrosis and inflammation in DFU

**Figure 6:**
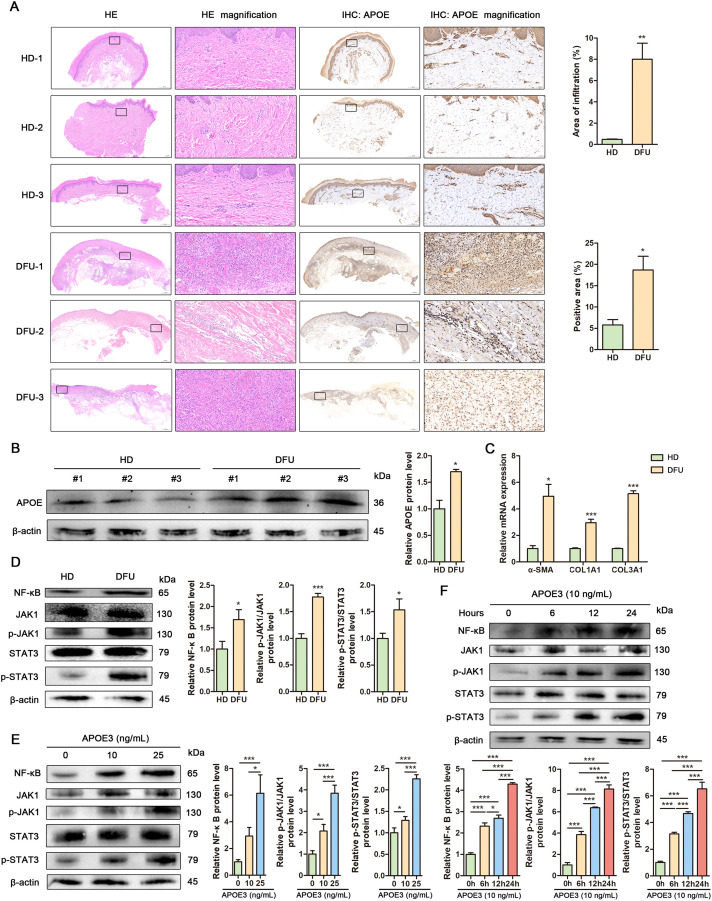
APOE+ fibroblasts promote fibrosis and inflammation in DFU. **(A)** Representative images showing histological differences between human healthy donor (HD) and diabetic foot ulcer (DFU) skin tissues assessed by HE staining (left) and APOE expression visualized by IHC (right). Insets display magnifications of different regions in the skin. Scale bar in the original images: 500 μm, 2000 μm. Scale bar in the magnified images: 50 μm. Upper: Cellular infiltration was quantified in HE-stained sections by measuring the percentage of infiltrated area. Lower: APOE expression was quantified through positive area percentage analysis of IHC staining. **(B)** Western blot analysis and quantitative comparison of APOE protein in human HD and DFU skin tissues. **(C)** RT-qPCR analysis of mRNA levels of α-SMA, COL1A1 and COL3A1 in human HD and DFU skin tissues. **(D)** Western blot analysis and quantitative comparison of the NF-κB and JAK1/Stat3 signaling pathways in human HD and DFU skin tissues. **(E)** Western blot analysis and quantitative comparison of the NF-κB and JAK1/Stat3 signaling pathways in human fibroblasts treated with APOE3 at concentrations of 10 ng/mL or 25 ng/mL. **(F)** Western blot analysis and quantitative comparison of the NF-κB and JAK1/Stat3 signaling pathways in human fibroblasts treated with APOE3 at a concentration of 10 ng/mL for 6 h, 12 h or 24 h. Data are presented as mean ± SD from three independent biological replicates (n = 3). *p< 0.05, ***p < 0.001.

To investigate fibroblast-related pathological changes, we analyzed biospecimens from healthy donors (HD) and DFU patients. Hematoxylin and eosin (HE) staining revealed disrupted skin tissue structure and significant inflammatory cell infiltration in DFU samples compared to HD. Immunohistochemical staining confirmed increased APOE expression in DFU samples compared to HD controls (**Figure 6A**), further supported by elevated APOE protein levels in Western blot analysis (**Figure 6B**). Fibrosis, a hallmark of DFU, was evidenced by upregulation of fibrosis-related genes (α-SMA, COL1A1, and COL3A1) in DFU skin samples (**Figure 6C**). To strengthen our fibrosis analysis, we conducted Masson’s trichrome staining and Picrosirius red staining. These results corroborate our gene expression data, showing increased collagen deposition and disorganized fiber structure in DFU skin tissues compared to HD controls (**Supplementary Figure S2A**). Quantitative analysis of collagen content via hydroxyproline assay showed increased collagen levels in DFU skin tissues compared to HD controls (**Supplementary Figure S2B**). Additionally, inflammation-related signaling pathways, including JAK/STAT3 and NF-κB, were activated in DFU tissues, suggesting their involvement in DFU-associated inflammation (**Figure 6D**). Pro-inflammatory cytokines including TNF-α, IL-6, IL-1β, showed significant elevation in DFU skin tissues compared to HD controls (**Supplementary Figure S3**). To assess APOE’s role in DFU inflammation, human fibroblasts were treated with recombinant APOE3 and analyzed by Western blot. APOE3 activated NF-κB and JAK/STAT3 signaling in dose- and time-dependent manners (**Figures 6E, F**). These findings highlight APOE+ fibroblasts as key contributors to fibrosis and inflammation in DFU.

### 2.7 Human fibroblasts exhibit elevated APOE expression, fibrosis and inflammation under high glucose conditions.

**Figure 7:**
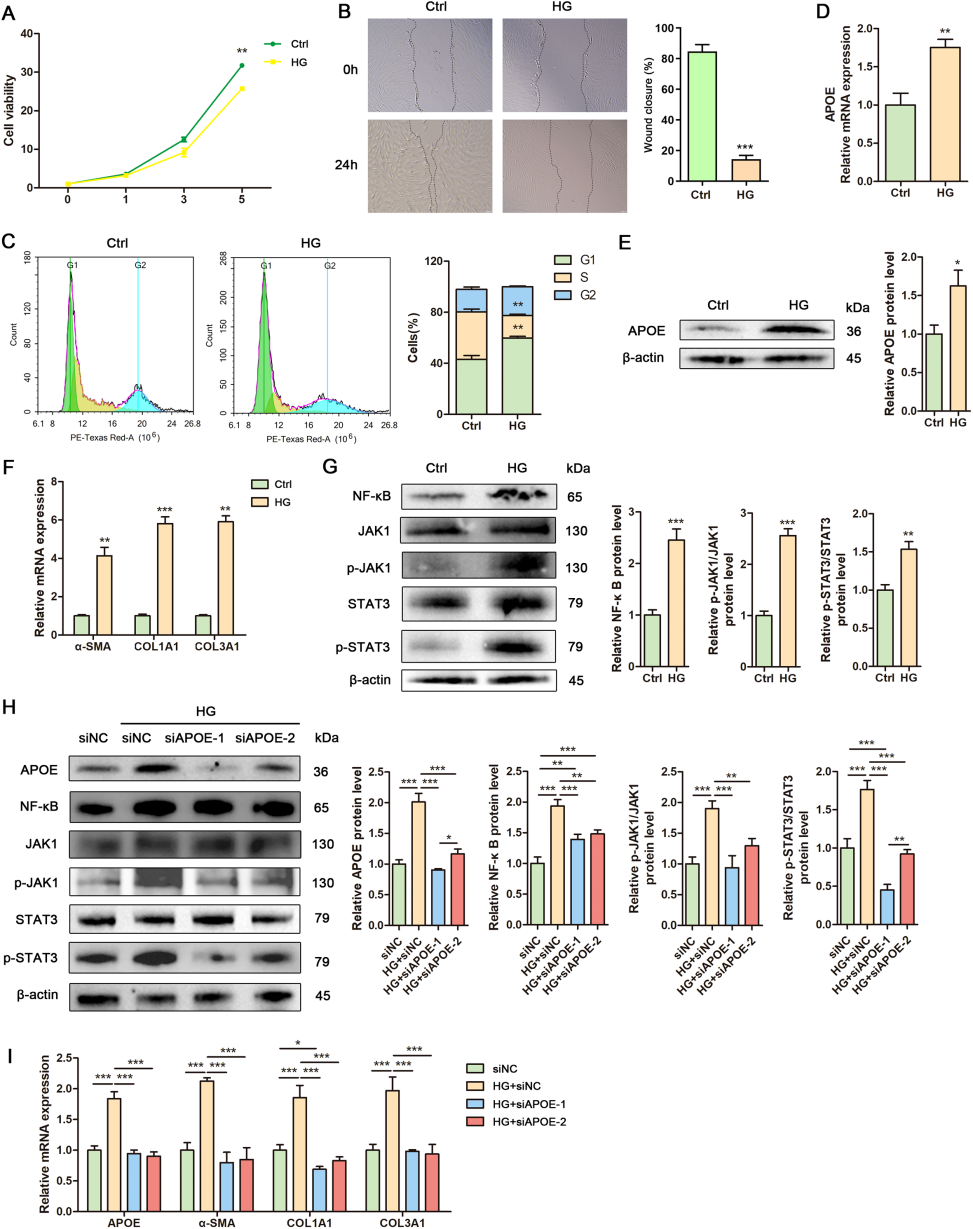
Human fibroblasts exhibit elevated APOE expression, fibrosis and inflammation under high glucose conditions. **(A)** Growth curve of human fibroblasts treated with high glucose. **(B)** Representative images and quantitative assessment of scratch wounds of human fibroblasts treated with or without high glucose at 0h and 24 h. **(C)** Flow cytometry analysis and quantitative assessment of cell cycle in human fibroblasts treated with or without high glucose. **(D)** RT-qPCR analysis of APOE mRNA level in human fibroblasts treated with or without high glucose. **(E)** Western blot analysis and quantitative comparison of APOE protein in human fibroblasts treated with or without high glucose. **(F)** RT-qPCR analysis of mRNA levels of α-SMA, COL1A1 and COL3A1 in human fibroblasts treated with or without high glucose. **(G)** Western blot analysis and quantitative comparison of the NF-κB and JAK1/Stat3 signaling pathways in human fibroblasts treated with or without high glucose. **(H)** Western blot analysis and quantitative comparison of the NF-κB and JAK1/Stat3 signaling pathways in human fibroblasts treated with control siRNA (siNC) or target siRNAs (siAPOE-1, siAPOE-2) under high glucose conditions. **(I)** RT-qPCR analysis of mRNA levels of APOE, α-SMA, COL1A1 and COL3A1 in human fibroblasts treated with control siRNA (siNC) or target siRNAs (siAPOE-1, siAPOE-2) under high glucose conditions. Data are presented as mean ± SD from three independent biological replicates (n = 3). *p < 0.05, **p < 0.01, ***p < 0.001.

To explore APOE as a potential therapeutic target for DFU, we established a high glucose-induced cell model, a well-characterized system for studying diabetic wounds. High glucose significantly inhibited fibroblast proliferation, as shown by the cell growth curve (**Figure 7A**). Additionally, Annexin V-FITC/PI staining revealed comparable apoptosis rates between control and high glucose-treated fibroblasts at day 1, day 3 and day 5 (**Supplementary Figure S4**), demonstrating that high glucose inhibits fibroblast proliferation without cytotoxic effects. Scratch assays revealed reduced fibroblast migration under high glucose conditions (**Figure 7B**). Flow cytometry analysis demonstrated cell cycle arrest in fibroblasts exposed to high glucose (**Figure 7C**). RT-qPCR and Western blot confirmed elevated APOE expression in fibroblasts under high glucose conditions (**Figures 7D, E**). Additionally, high glucose induced fibrosis and inflammation in fibroblasts (**Figures 7F, G**). These findings indicate that high glucose upregulates APOE expression and promotes fibrosis and inflammation in fibroblasts, suggesting that APOE overexpression may drive DFU progression. Knockdown of APOE expression using siRNAs in high glucose-treated fibroblasts attenuated the activation of NF-κB and JAK/STAT3 signaling pathways and downregulated fibrosis-related genes (α-SMA, COL1A1, and COL3A1) induced by high glucose (**Figures 7H, I**). These results demonstrate that APOE mediates high glucose-induced pro-fibrotic and inflammatory responses in fibroblasts.

## 3 Discussion

Diabetic foot ulcers (DFU) are a prevalent and severe complication of long-term, poorly managed diabetes. DFU is defined as a break in the epidermis and part of the dermis in individuals with diabetes. Tissue inflammation and fibrosis play critical roles in the development and progression of DFU (12). However, the precise cellular and molecular mechanisms driving these processes remain unclear.

Recent studies have enhanced our understanding of fibroblasts as a morphologically and functionally heterogeneous cell population (13). Here, we analyzed scRNA-Seq data from tissue samples of DFU patients, recovered DFU patients, healthy individuals, and diabetic patients without DFU to explore cellular heterogeneity in DFU. We identified twelve distinct cell clusters: fibroblasts, macrophages, smooth muscle cells, T-NK cells, keratinocytes, endothelial cells, B-plasma cells, cDC2, proliferating cells, mast Cells, melanocytes, and schwann cells. GO-GSEA enrichment analysis revealed significant clustering of fibroblast subpopulations in extracellular matrix organization, encapsulating structure organization, collagen fibril organization, and extracellular structure organization, suggesting their active involvement in these processes.

Dermal fibroblasts were identified as a highly diverse and heterogeneous population with distinct functional roles in diabetic wound healing (14, 15). Based on marker genes, we classified fibroblasts primarily based on their marker genes into the following categories: C0 APOE+ fibroblasts; C1 AQP1+ fibroblasts; C2 TNC+ fibroblasts; C3 NR2F2+ fibroblasts; C4 TNN+ fibroblasts. These marker genes suggest diverse biological functions relevant to wound healing and diabetic foot ulcer (DFU) pathology.

For instance, the APOE-high subgroup may be involved in lipid metabolism and immune modulation, influencing local inflammatory responses (16). The AQP1-expressing fibroblasts likely contribute to water transport and tissue hydration (17), crucial for maintaining wound microenvironment. TNC (18) and TNN (19) both extracellular matrix-related genes, imply roles in matrix remodeling and structural integrity. NR2F2, a transcription factor, may regulate fibroblast differentiation and vascular interactions, affecting tissue repair dynamics (20).

Together, these marker gene profiles reflect the functional heterogeneity of fibroblasts in DFU wounds. Understanding how these subpopulations interact and contribute to processes like inflammation, matrix deposition, and regeneration can provide novel insights for targeted therapeutic strategies. The transcription factor analysis revealed that the C0 fibroblast subpopulation is closely associated with the M2 regulon cluster, which is significantly enriched in DFU samples. Many of the transcription factors identified have been reported to regulate key cellular processes including proliferation, migration, and differentiation of fibroblasts. For example, NR2F2 has been shown to modulate fibroblast activation and extracellular matrix remodeling, which are critical steps in wound healing (21).

In the context of DFU, dysregulation of these transcription factors may impair normal fibroblast function, contributing to chronic wound persistence. They may affect fibroblast proliferation by regulating cell cycle genes, influence migration by modulating cytoskeletal and adhesion molecule expression, and alter differentiation by controlling lineage-specific gene expression. Further experimental validation is required to elucidate the precise mechanisms through which these transcription factors regulate fibroblast behavior in diabetic wounds. Validation at protein levels confirmed elevated APOE+ fibroblast expression in DFU skin tissues compared to HD skin tissues. APOE, a major cholesterol carrier that facilitates lipid transport and injury repair (22), was further analyzed for metabolic pathway analysis. We found that APOE+ subpopulation showed strong associations with fat cell differentiation and regulation of epithelial cell proliferation. In addition, pseudotime analysis indicated an intermediate differentiation state of APOE+ fibroblasts.

The Drug Metabolism–Cytochrome P450 pathway was downregulated in tissue samples from recovered diabetic foot patients compared to those from the disease group, suggesting that the APOE+ subpopulation might influence the onset and progression of diabetes through this metabolic pathway. The Drug Metabolism-Cytochrome P450 enzyme family plays a critical role in the metabolism of endogenous and exogenous compounds, including drugs, lipids, and toxins. Dysregulation of this pathway may contribute to impaired detoxification, increased oxidative stress, and altered cellular metabolism, which are known to exacerbate tissue damage and delay wound healing in diabetic patients. Moreover, altered Drug Metabolism-Cytochrome P450 activity has been reported to affect inflammatory responses and angiogenesis, both crucial processes in ulcer development and repair (18). Besides the Drug Metabolism-Cytochrome P450 pathway, two other metabolic pathways identified in this study are closely associated with the chronic inflammation and metabolic dysregulation observed in diabetic foot ulcers (DFUs), indicating their potential involvement in disease progression. Firstly, glutathione metabolism was found to be upregulated in multiple cell types, especially in macrophages and fibroblasts. Glutathione is a key intracellular antioxidant that helps neutralize reactive oxygen species (ROS) and maintain redox homeostasis. Its activation may reflect a protective adaptation to the persistent oxidative stress present in chronic DFU wounds (23). Oxidative stress is a recognized contributor to impaired wound healing in diabetes. Secondly, the upregulation of pyruvate metabolism in immune cells suggests a metabolic shift toward aerobic glycolysis, a hallmark of chronic inflammatory conditions and cancer (24). This reprogramming may impair energy efficiency and amplify inflammatory cytokine production, thereby contributing to delayed tissue repair and chronic non-healing phenotypes. These metabolic alterations may collectively disrupt cellular energy homeostasis and biosynthesis, further impairing fibroblast function and wound healing capacity. Future studies focusing on the integration of these metabolic changes may provide deeper insight into DFU pathogenesis and reveal novel therapeutic targets.

In DFU, cells act as both signal senders (releasing cytokines/ligands) and receivers (responding to ligands) (25, 26). In this study, we identified the C0 fibroblast subpopulation as playing a key role in fibroblast differentiation. Based on the CellChat analysis, we further explored the interactions between the C0 subpopulation and other cell types in the diabetic foot ulcer microenvironment, especially endothelial cells and smooth muscle cells. The C0 subpopulation may regulate local angiogenesis, immune responses, and extracellular matrix remodeling through multiple cell-cell communication signaling pathways, thereby promoting wound healing and tissue repair. Previous studies have shown that interactions between fibroblasts and endothelial cells are crucial for angiogenesis and wound healing (27), while smooth muscle cells also contribute to vascular stability and tissue repair processes (28). Therefore, a deeper investigation into the cell communication mechanisms of the C0 subpopulation could provide valuable insights into the pathophysiology of the diabetic foot ulcer microenvironment and offer potential targets for therapeutic intervention. Fibroblast interactions with monocytes/macrophages in inflammation and wound healing (29, 30), as well as strong signaling between fibroblasts and endothelial cells during diabetic wound healing (31), highlight the importance of intercellular communication in DFU progression. Our findings suggest that APOE+ fibroblasts exhibit strong interactions with keratinocytes, and may play a significant role in the FGF signaling pathway, which promotes angiogenesis and accelerates wound healing (32). Collectively, these data implicate APOE+ fibroblasts as critical contributors to DFU healing.

Fibrosis, characterized by excessive deposition of extracellular matrix (ECM) proteins, is a key pathological feature of diabetic wounds (15). Fibroblasts play a critical role in diabetes and its complications, with fibrotic mechanisms potentially involving direct activation of permanent fibroblasts (33). Recent studies highlight pro-inflammatory fibroblasts as key contributors to DFU progression, expressing various inflammatory mediators (34). Chronic inflammation and hyperglycemia in diabetic skin drive injury-related gene expression in fibroblasts, including a COL7A1-expressing subpopulation (8). NF-κB, activated by cytokine receptors and Toll-like receptor 4 (TLR4), regulates inflammation-related genes such as TNFα and IL-6 (35). The JAK/STAT pathway, a major signaling route for cytokines and growth factors, is crucial for inflammation-related gene transcription (36). NF-κB and STAT3 interactions are vital in mediating communication between cancer and inflammatory cells (37). In this study, upregulation of NF-κB and JAK/STAT3 was revealed in DFU fibroblasts compared to normal fibroblasts. Additionally, fibrosis-related genes and pathways, including α-SMA, COL1A1, and COL3A1 (38), were significantly elevated in DFU fibroblasts. These findings suggest that APOE+ fibroblasts exhibit enhanced activation of fibrosis- and inflammation-related signaling pathways, underscoring their potential role in DFU progression.

APOE, a classic component of lipoprotein complexes, is traditionally known for its role in lipid metabolism through binding to LDLR family members, including LDLR and LDLR-related protein 1 (LRP1) (39). Improving the lipidated state of APOE can mitigate APOE-associated central nervous system impairments (40). APOE reduces inflammation by neutralizing LPS (41) and regulating NF-κB signaling (42) in macrophages, thereby limiting inflammatory cytokine production and atherosclerosis. Elevated APOE expression in fibroblasts and macrophages promotes cartilage degeneration in osteoarthritis (OA), while APOE inhibition alleviates OA progression (43). Despite these advances, the role of APOE in human skin fibroblasts remains poorly understood. Our findings suggest that APOE promotes fibrosis and inflammation in human fibroblasts, potentially via NF-κB and JAK/STAT3 signaling pathways. Previous studies indicate that high glucose (HG) induces fibroblast senescence in diabetic wounds (44). In HG environments, excessive reactive oxygen species (ROS) activate STING signaling by triggering mitochondrial DNA (mtDNA) release into the cytoplasm, promoting pro-inflammatory macrophage polarization and exacerbating endothelial cell dysfunction (45). Notably, APOE2-expressing myeloid cells exhibit elevated intracellular cholesterol due to impaired efflux, driving inflammasome activation and myelopoiesis, while APOE4-expressing cells promote inflammation through oxidative stress, independent of inflammasome signaling (46). Our results reveal that HG inhibits fibroblast proliferation, migration, and cell cycle progression. Furthermore, HG elevates APOE expression and enhances fibrosis and inflammation in fibroblasts. Knockdown of APOE expression in high glucose-treated fibroblasts attenuated the activation of NF-κB and JAK/STAT3 signaling pathways and downregulated fibrosis-related genes induced by high glucose. These results demonstrate that APOE mediates high glucose-induced pro-fibrotic and inflammatory responses in fibroblasts, suggesting that APOE+ fibroblasts contribute to DFU progression. Local inhibition of APOE in wounds may thus represent a novel therapeutic strategy for DFU treatment. While our in vitro data suggest high glucose may prime fibroblasts for pro-fibrotic and pro-inflammatory responses, further in vivo validation is needed to assess this mechanism in diabetic wound contexts. Future studies should investigate the specific receptor mediating APOE’s effects on DFU fibroblasts.

This study has several limitations that should be considered. The DFU cohort size, though appropriate for initial single-cell characterization, may constrain the detection of subtle biological effects and limit generalizability across this clinically diverse patient population. Demographic disparities between patients and controls, combined with unstratified disease severity, represent additional variables that could influence the interpretation of cellular profiles. The cross-sectional nature of our dataset also precludes analysis of temporal changes during ulcer progression. These constraints reflect the inherent challenges of obtaining foot tissue biopsies from DFU patients, where ethical considerations and clinical practicality necessarily limit sample availability. To address these limitations, we implemented complementary experimental validations through in vitro models to ensure robust biological concordance. Nonetheless, our work establishes the detailed single-cell atlas of DFU fibroblasts, uncovering fundamental aspects of cellular heterogeneity while identifying APOE as a mechanistically plausible therapeutic target. Subsequent studies incorporating larger, demographically balanced cohorts with longitudinal sampling will be crucial to verify these observations and explore clinically relevant patient stratifications.

## 4 Materials and methods

### 4.1 Human specimens

Biospecimens of DFU were collected from patients experiencing the debridement of diabetic wounds. Biospecimens of HD were collected from patients experiencing the removal of pigmented nevi. Discarded skin specimens were obtained from Department of Burn and Plastic Surgery, Seventh People’s Hospital Affiliated to Shanghai University of Traditional Chinese Medicine. All protocols involving human subjects were reviewed and approved by the Institutional Review Board of Shanghai seventh People’s Hospital (2024-7th-HIRB-016). All procedures were carried out in accordance with guidelines set forth by Declaration of Helsinki. Written informed consent was obtained from all participants.

### 4.2 Data preprocessing and quality control

The scRNA-seq data of skin biopsies from non-DM subjects, DM patients with no DFU, and DM patients with DFU (Healers and Non-healers) were obtained from the Gene Expression Omnibus (GEO) database. GEO accession number is GSE165816. The raw single-cell RNA sequencing (scRNA-seq) data were initially processed to generate a count matrix. Subsequent analysis was conducted using the Seurat package (v4.3.0) within the R programming environment (v4.2.0), following established methodologies. To ensure high-quality data and remove doublets, the DoubletFinder package (v2.0.3) was employed. Quality control measures involved retaining cells with a feature count between 300 and 7,500, a total count between 500 and 100,000, and mitochondrial gene expression below 20% of total gene expression per cell. Additionally, cells exhibiting erythroid gene expression exceeding 5% of total expressed genes were filtered out. Normalization techniques were applied to mitigate biases related to library size and cell-specific differences. Highly variable genes were identified based on expression variance, with the top 2000 genes selected for further processing. To address batch effects across samples, the Harmony R package (v0.1.1) was utilized, incorporating the ComBat algorithm to minimize technical biases arising from different experimental batches. Furthermore, the removeBatchEffect function from the limma package was applied to enhance batch effect correction. Clustering analysis was performed using the top 30 principal components (PCs), with Uniform Manifold Approximation and Projection (UMAP) used for visualization in a two-dimensional space. Cell type annotation was conducted based on Seurat’s built-in tools, previous literature, and known marker genes from the CellMarker database. Differential expression analysis was performed using the “FindAllMarkers” function with log2 fold change (FC) > 0.5 and an adjusted p-value < 0.01 as selection criteria. The proportion of each cell type was then determined by analyzing their distribution across different clusters. To ensure data quality, we applied stringent quality control (QC) criteria to the raw single-cell expression matrix. Specifically, cells were retained if they had between 200 and 6,000 detected genes (nFeature_RNA), fewer than 30,000 total transcripts (nCount_RNA), and less than 10% mitochondrial gene expression (percent.mt) (47).

### 4.3 Cell type annotation

During the annotation of cell subpopulations, we employed a multi-step strategy for cell type naming. First, we performed differential gene expression analysis using Seurat’s FindAllMarkers function to identify cluster-specific marker genes. Based on the known biological functions of these genes and their reported roles in the literature, we assigned preliminary cell type identities. In addition, we cross-referenced these markers with curated cell type marker databases such as CellMarker to validate and refine our annotations. For clusters with ambiguous marker profiles, we further conducted functional enrichment analyses including Gene Ontology (GO), Kyoto Encyclopedia of Genes and Genomes (KEGG) and Gene Set Enrichment Analysis (GSEA) to support the annotation and enhance both its accuracy and biological interpretability.

### 4.4 InferCNV

To evaluate the genomic stability of fibroblast subpopulations and exclude malignant features, we applied the inferCNV package (v1.10.1) for CNV inference. Epithelial cells from healthy control samples were used as the reference. Raw counts were log-transformed and denoised using default parameters, including a sliding window of 100 genes and a cutoff of 1.0. Clustering was performed using hierarchical methods based on CNV patterns.

### 4.5 Bulk RNA-sequencing analysis

Differential expression analysis was performed separately for healed and non-healed samples using the DESeq2 R package. Genes with |logFC| > 2 and a p-value < 0.05 were considered significantly differentially expressed. The identified genes were further analyzed for GO, KEGG, and GSEA using the clusterProfiler package.

### 4.6 Pathways and systems biology analysis

To gain insights into cell-type-specific molecular mechanisms underlying wound healing, pathway enrichment and systems biology analysis were conducted. Transcripts significantly dysregulated in different cell populations were compared between healed and non-healed samples. Ingenuity Pathway Analysis (IPA 9.0, Qiagen) was utilized for pathway analysis, with details available on the Ingenuity Systems website (http://www.ingenuity.com). Upstream transcriptional regulators were identified through systems biology analysis to determine significantly activated or inhibited regulators. The statistical significance of transcriptional regulator activity was assessed using a one-tailed Fisher’s exact test, with regulators meeting the criteria of p-value < 0.01 and an absolute z-score > 2 considered significant.

### 4.7 Metabolic pathway analysis

To comprehensively explore metabolic alterations across different biological states, metabolic pathway analysis was conducted through a combination of computational methods and database-driven approaches. Differentially expressed genes (DEGs) derived from transcriptomic datasets were annotated to established metabolic pathways using resources such as the KEGG. Functional enrichment analysis was performed utilizing the clusterProfiler R package, with statistically significant pathways identified based on an adjusted p-value threshold of < 0.05. In parallel, metabolomics data were examined using MetaboAnalyst 5.0, where metabolites showing significant variation (p-value < 0.05, VIP score > 1) underwent pathway overrepresentation and network topology analysis. The relative impact of each metabolic pathway was determined by assessing metabolite importance and their connectivity within the network.

To identify transcription factors potentially regulating gene expression in specific cell populations, we performed motif enrichment analysis using the cisTarget pipeline. This approach utilizes precompiled motif rankings derived from databases such as JASPAR, HOCOMOCO, and CIS-BP, integrated within the cisTarget resource. Motif enrichment was calculated based on the presence of conserved binding motifs in the regulatory regions of differentially expressed genes. This method allows for robust and reproducible inference of key TFs driving cell-type-specific gene regulatory programs.

### 4.8 Ro/e ratio analysis

To identify preferential enrichment of fibroblast subpopulations across different clinical conditions, we calculated the Ro/e ratio (observed/expected ratio) for each cluster in each group. This metric quantifies whether a given cell type is overrepresented (Ro/e > 1) or underrepresented (Ro/e < 1) relative to the total fibroblast pool, adjusting for sample composition. Statistical significance was assessed using a hypergeometric test.

### 4.9 Differential expression and functional enrichment analysis

Cell-type-specific DEGs were identified using the “FindAllMarkers” function in Seurat, applying the Wilcoxon rank-sum test with default parameters. Genes were selected based on logFC > 0.25 and detection in at least 25% of cells within each cluster. Functional enrichment analysis of DEGs was performed using the clusterProfiler package (v0.1.1) to explore associated biological processes.

### 4.10 Subpopulation analysis and functional enrichment

Differential expression analysis across subpopulations was carried out using the “FindAllMarkers” function in Seurat, employing the Wilcoxon rank-sum test. The selection criteria included only.pos = TRUE, min.pct = 0.25, and logFC.threshold = 0.25. GO-BP enrichment analysis was performed on the identified DEGs using the clusterProfiler package to explore their biological functions.

### 4.11 GSEA

Differential gene analysis was conducted using GSEA 4.1.0 on an annotated expression matrix from the GEO database. The analysis was performed with 1,000 permutations, with phenotype labels set as PI versus normal. The permutation type was designated as phenotype-based, and the top 2000 DEGs between the PI and normal groups were identified. The resulting gene sets were intersected with potential targets to validate significant genes.

### 4.12 Trajectory analysis

To examine differentiation trajectories of fibroblast subpopulations, an integrated approach utilizing three computational tools was applied. First, the cytoTRACE algorithm assessed cellular differentiation potential and stemness. Next, the Monocle R package (v2.24.0) reconstructed differentiation trajectories using the DDRTree algorithm. Finally, the Slingshot package (v2.6.0) inferred lineage relationships by constructing a minimum spanning tree (MST). Gene expression changes along differentiation trajectories were estimated using the getCurves function.

### 4.13 Cell-cell communication analysis

Intercellular communication within DFU tissues was analyzed using the CellChat R package (v1.6.1). Ligand-receptor interactions were inferred using CellChatDB. human as a reference database, providing insights into signaling networks and intercellular interactions. Ligands expressed in ≥5% of sender populations and receptors in ≥5% of receiver populations were included, enabling detailed characterization of signaling interplay across cell types. Prior to analysis, low-quality cells were filtered based on standard QC metrics (e.g., number of genes detected <200 or mitochondrial gene percentage >20%). The normalized and scaled gene expression matrix from Seurat was imported into CellChat. The minimum number of cells per group was set to 10 to ensure robust inference. Pathway-specific communication probabilities were inferred and visualized using netVisual_circle and netAnalysis_signalingRole functions.

### 4.14 Isolation and culture of human fibroblasts

Skin specimens were stored in sterile PBS on ice, then incubated in 5 mg/ml Dispase II (Thermo Fisher, USA) in HBSS (Gibco, USA) overnight at 4°C. The epidermis was removed, and tissues were minced, washed, and digested with 0.1% collagenase I (Sigma-Aldrich, USA) in DMEM (Gibco, USA) for 1 h at 37°C. After centrifugation, the cell pellet was resuspended, filtered through a 70 µm cell strainer and plated in DMEM with 10% FBS (Gibco, USA) and 1% PSA (Gibco, USA). Non-adherent cells were removed by medium replacement after 24–48 hours. Cells were cultured to 80% confluence, detached with 0.05% Trypsin/EDTA, and resuspended in complete medium.

### 4.15 Immunohistochemistry

Formalin-fixed paraffin-embedded tissue was deparaffinized, rehydrated through graded alcohols, and subjected to heat-induced antigen retrieval. The tissue was blocked with 3% BSA for 1 hour, followed by overnight incubation at 4°C with primary antibody against APOE (1:1000, Affinity, China) in 3% BSA. A secondary antibody was then applied for 1 hour at room temperature. For DAB staining, 3,3’-diaminobenzidine was used as the chromogen, and hematoxylin was applied for counterstaining.

### 4.16 *Ex vivo* stimulation of human fibroblasts

Cells were treated with human recombinant APOE3 protein (MedChemExpress, China) at 10 ng/mL or 25 ng/mL for 24 hours, or at 10 ng/mL for 6, 12, or 24 hours. Separately, cells were cultured in normal glucose (5.5 mM) or high glucose (25 mM) medium for 24 hours.

### 4.17 Western blot

Tissue or cell extracts were separated by SDS-PAGE, and proteins were transferred to a nitrocellulose membrane. The membrane was incubated with primary antibodies: anti-APOE (1:1000, Affinity, China), anti-NF-κB (1:1000, CST, USA), anti-p-JAK1 (1:1000, CST, USA), anti-JAK1 (1:1000, CST, USA), anti-p-STAT3 (1:1000, CST, USA), anti-STAT3 (1:1000, CST, USA), and anti-β-actin (1:10000, Abclone, China). Immunoreactive bands were visualized using a Servicebio (China) scanning system. Quantitative analysis was performed using ImageJ software (NIH, USA).

### 4.18 RNA extraction and RT-qPCR

Total RNA was extracted using the Cell/Tissue Total RNA Kit (NCMbiotech, China) and reverse-transcribed into cDNA using the PrimeScript RT Master Mix Kit (TaKaRa, Japan) with oligo-dT primers. RT-qPCR was performed using TB Green Premix Ex Taq (TaKaRa, Japan), with specific primers obtained from Servicebio. Relative gene expression was calculated using the 2^−ΔΔCT^ method. Specific primers are as follows: α-SMA forward 5’-CAA TGT CCT ATC AGG GGG CAC-3’and reverse 5’-CGG CTT CAT CGT ATT CCT GTT-3’; COL1A1 forward 5’-CCC CTG GAA AGA ATG GAG ATG-3’and reverse 5’- AGC TGT TCC GGG CAA TCCT-3’; COL3A1 forward 5’-CCC CGT ATT ATG GAG ATG AACC-3’and reverse 5’- CCA TCA GGA CTA ATG AGG CTT TC-3’; GAPDH forward 5’- GGA AGC TTG TCA TCA ATG GAA ATC-3’ and reverse 5’- TGA TGA CCC TTT TGG CTC CC-3’.

### 4.19 Cell viability assay

Cell viability was evaluated using the CCK-8 assay (Beyotime, China) following the manufacturer’s protocol. Absorbance at 450 nm was measured using a microplate reader (Flexstation 3, USA), and results were expressed as a percentage relative to control cells.

### 4.20 Scratch assay

A straight scratch was made in the cell monolayer using a 200 µl pipette tip. After washing once with media to remove debris, cells were incubated in fresh medium. Wound closure percentage was calculated using the standard formula ((T0 - T24)/T0)) × 100.

### 4.21 Cell cycle flow cytometric analysis

Cells were harvested and fixed in 70% ethanol overnight at 4°C. Fixed cells were washed and stained with RNase A and PI (KeyGEN, China) for 30 minutes at room temperature in the dark. Cell cycle distribution was analyzed using a NovoCyte Advanteon flow cytometer (Agilent, USA).

### 4.22 siRNA transfection

Cells at 60-70% confluence were transfected with 50 nM siRNA (Hanbio, China) using Lipofectamine 3000 (Thermo Fisher, USA). Medium was replaced after 6 h, and cells were harvested 48–72 h post-transfection for analysis. siNC: sense 5’- UUC UCC GAA CGU GUC ACG UTT -3’ and antisense 5’- ACG UGA CAC GUU CGG AGA A TT -3’; siAPOE-1: sense 5’- GCU GAU GGA CGA GAC CAU GAA TT -3’and antisense 5’- UUC AUG GUC UCG UCC AUC AGC TT -3’; siAPOE-2: sense 5’- GCC UCA AGA GCU GGU UCG AGC TT -3’and antisense 5’- GCU CGA ACC AGC UCU UGA GGC TT -3’.

### 4.23 Statistical analysis

The data represent the mean ± standard deviation (SD) of three independent biological replicates (n = 3). Differences between two groups were assessed using a *t*-test, while multiple comparisons were analyzed by one-way ANOVA (SPSS 16.0, Inc., USA). A *p*-value < 0.05 was considered statistically significant.

## Data Availability

The datasets presented in this study can be found in online repositories. The names of the repository/repositories and accession number(s) can be found in the article/**Supplementary Material**.
