# Supplementary figures and images for: Single-cell atlas of human skin implicates APOE pro-inflammatory signaling in diabetic foot ulcers
Source: Front Immunol. 2025 Jun 19;16:1591944. doi: 10.3389/fimmu.2025.1591944 (PMC12221912; doi:10.3389/fimmu.2025.1591944)

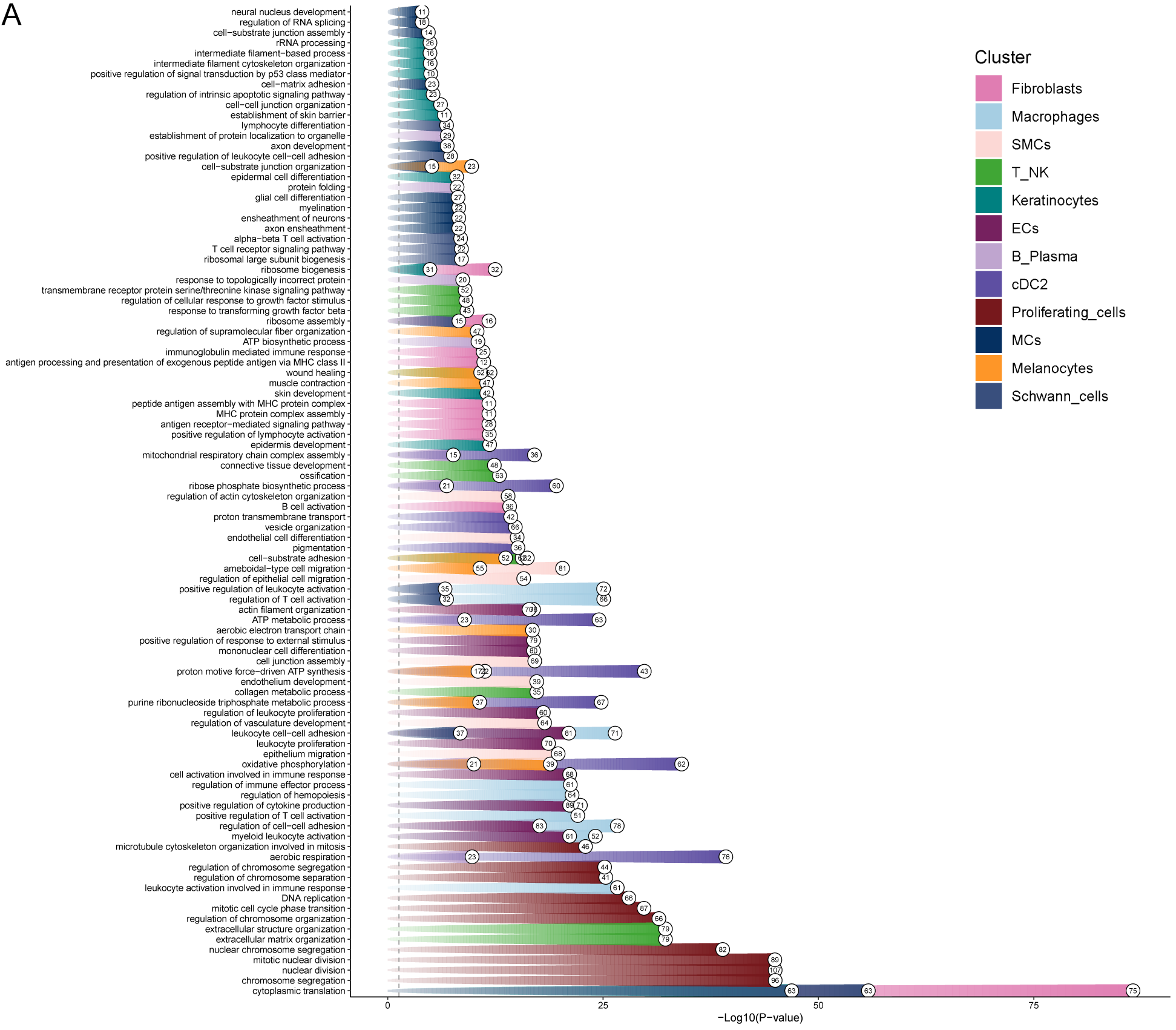

Supplement: Supplementary file 1 [file Image1.tif]

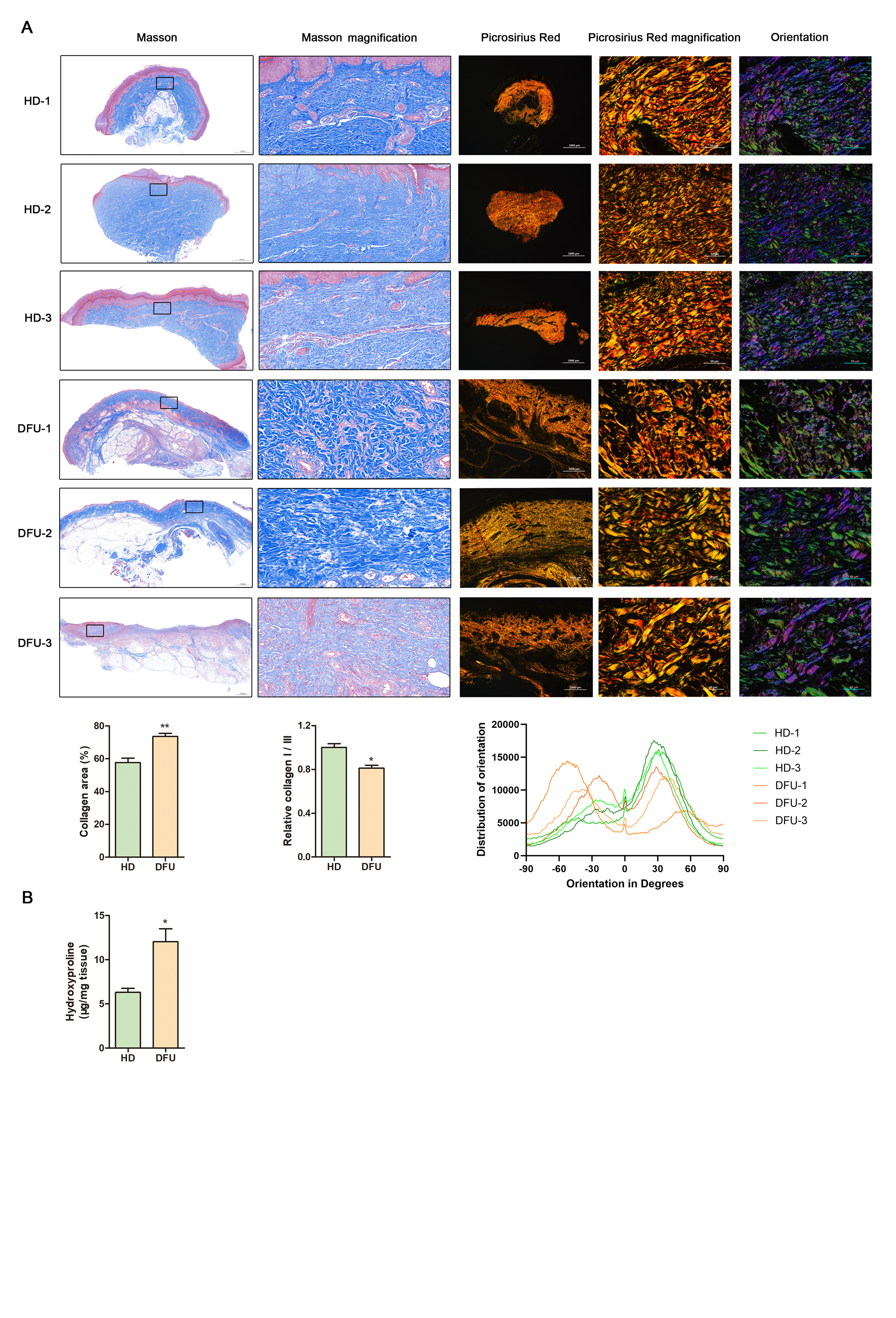

Supplement: Supplementary file 2 [file Image2.tif]

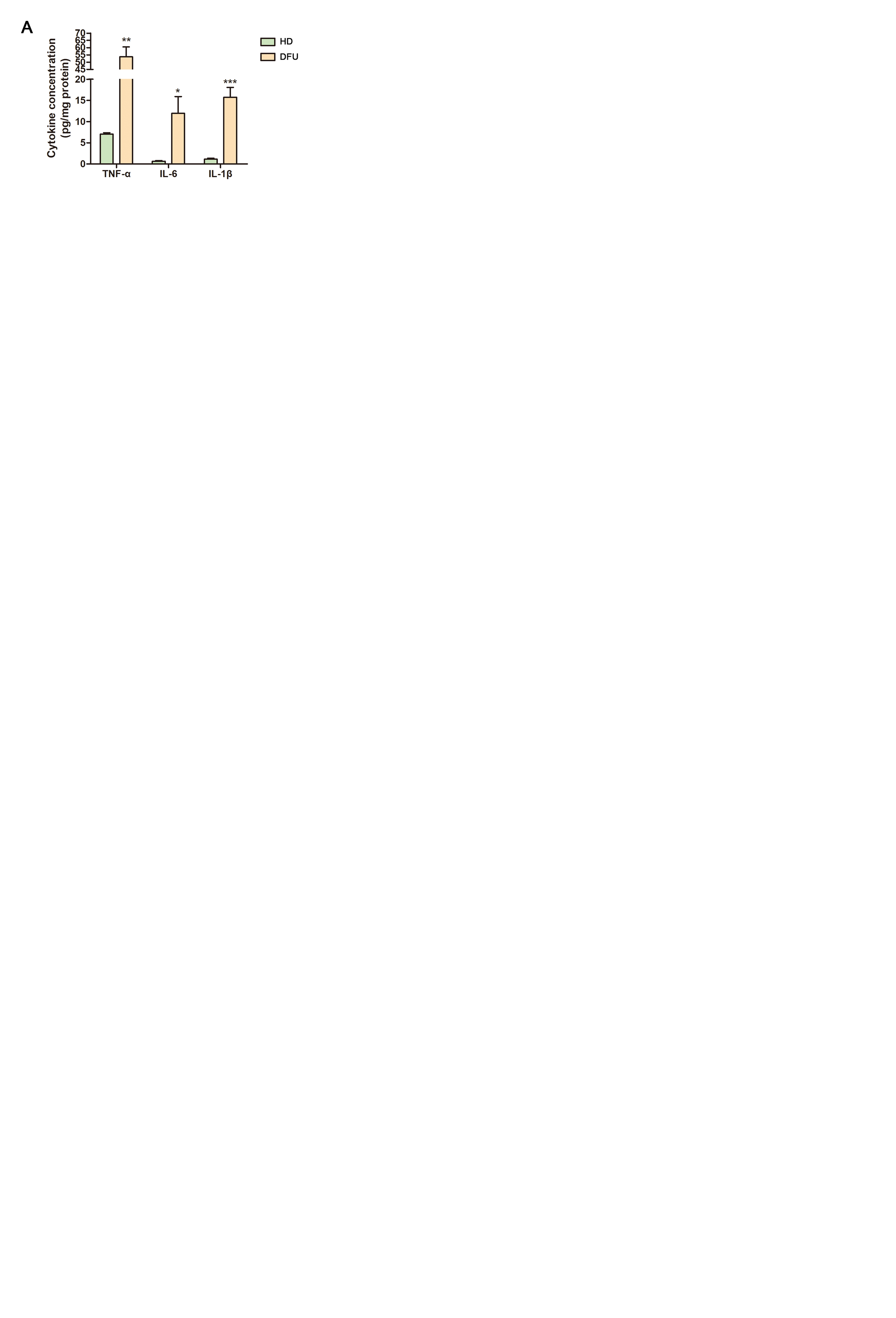

Supplement: Supplementary file 3 [file Image3.tif]

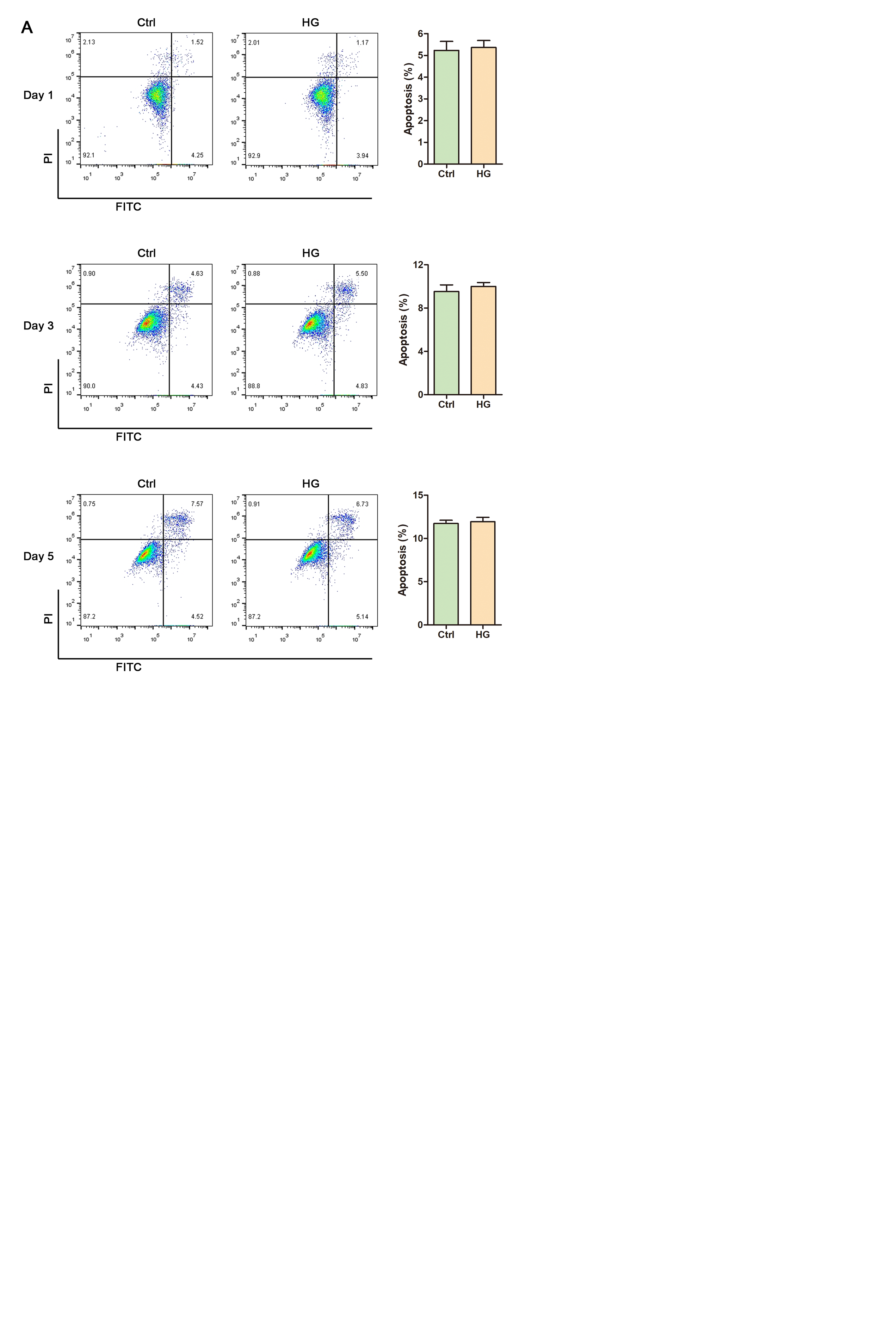

Supplement: Supplementary file 4 [file Image4.tif]
