# Supplementary material for: Single-cell atlas of human skin implicates APOE pro-inflammatory signaling in diabetic foot ulcers
Source: Front Immunol. 2025 Jun 19;16:1591944. doi: 10.3389/fimmu.2025.1591944 (PMC12221912; doi:10.3389/fimmu.2025.1591944)
Supplement: Supplementary file 5 [file DataSheet1.docx]

**Supplementary Materials and methods**

**Masson’s trichrome staining**

Formalin-fixed paraffin-embedded tissue was stained using Masson’s trichrome kit (Servicebio, China) to visualize collagen (blue) and muscle/cytoplasm (red). The collagen area percentage was calculated as the ratio of collagen-positive area (blue) to total tissue area ×100. Three randomly selected high-power fields (HPFs, ×200) per sample were analyzed using ImageJ software (NIH, USA).

**Picrosirius red staining**

Formalin-fixed paraffin-embedded tissue was incubated in 0.1% Picrosirius red (Servicebio, China) and analyzed under polarized light. Thick collagen fibers (Type I) appeared yellow/orange, while thin fibers (Type III) exhibited green. Collagen orientation was quantified in ImageJ using Orientation analysis.

**Hydroxyproline assay**

Tissue homogenates (15 mg in 150 μL H₂O) were subjected to alkaline hydrolysis (100°C, 3 h), followed by acid neutralization and centrifugation. Hydroxyproline content was determined through chloramine-T oxidation (60°C, 15 min) and spectrophotometric analysis at 560 nm. The assay was conducted following the manufacturer's (Servicebio, China) protocol. Hydroxyproline content was expressed as μg per mg of wet tissue weight (μg/mg tissue).

**ELISA**

Tissue lysates were analyzed for TNF-α, IL-6, and IL-1β using ELISA kits (Servicebio, China), following manufacturer’s instructions. Absorbance (450 nm) was measured and concentrations calculated against standard curves. Final cytokine levels were normalized to total protein content and expressed as pg per mg of protein (pg/mg protein).

**Apoptosis assay**

Cells were stained with Annexin V-FITC/PI (BD Biosciences, USA) and analyzed by flow cytometry to quantify apoptosis. The total apoptosis rate was calculated as the sum of early apoptotic (Annexin V-FITC+/PI-) and late apoptotic (Annexin V-FITC+/PI+) cell populations.

**Supplementary Figure legends**

**Figure S1 Functional activity landscape and subpopulation contributions.**

A. Stacked bar plots illustrate the relative contributions of distinct cell subpopulations to various biological processes. The y-axis represents enriched biological processes, while the x-axis indicates the relative activity or enrichment score of each process. Different colors correspond to specific cell subpopulations, reflecting their proportional involvement in each functional activity.

**Figure S2 Collagen deposition and disorganized fiber structure were observed in DFU skin tissues.**

A. Representative images of histological differences in collagen deposition and fiber organization between HD and DFU skin tissues. Left: Masson’s trichrome staining. Scale bar in the original images: 500 μm, 2000 μm. Scale bar in the magnified images: 50 μm. Right: Picrosirius red staining under polarized light. Scale bar in the original images: 1000 μm. Scale bar in the magnified images: 50 μm. Left: The collagen area percentage was quantified from Masson’s trichrome-stained sections using ImageJ software. Middle: The classification and spatial deposition of collagen I (yellow/orange) and collagen III (green) were analyzed using Picrosirius red staining under polarized light. Right: Collagen fiber orientation was quantified in ImageJ using Orientation analysis. 0°: horizontal alignment; 90°: vertical alignment.

B. Quantitative analysis of collagen content as measured by hydroxyproline assay between the HD and DFU skin tissues.

Data are presented as mean ± SD from three independent biological replicates (n = 3). **p* < 0.05, ***p* < 0.01.

**Figure S3 Pro-inflammatory cytokines showed significant elevation in DFU skin tissues.**

A. ELISA quantification of TNF-α, IL-6, IL-1β in HD and DFU skin tissues.

Data are presented as mean ± SD from three independent biological replicates (n = 3). **p* < 0.05, ***p* < 0.01, ****p* < 0.001.

**Figure S4 High glucose did not induce significant fibroblast cytotoxicity.**

A. Apoptosis rates in control and high glucose-treated fibroblasts at day 1, day 3 and day 5, as determined by Annexin V-FITC/PI staining.

Data are presented as mean ± SD from three independent biological replicates (n = 3).
